# Detection Efficiency of Ag Nanoparticle Labels for a Heart Failure Marker Using Linear and Square-Wave Anodic Stripping Voltammetry

**DOI:** 10.3390/bios12040203

**Published:** 2022-03-29

**Authors:** Nikhil Raj, Richard M. Crooks

**Affiliations:** Department of Chemistry, The University of Texas at Austin, 105 E. 24th Street, Stop A5300, Austin, TX 78712-1224, USA; nikhil.raj@austin.utexas.edu

**Keywords:** square-wave voltammetry, metalloimmunoassay, heart failure, point-of-care diagnostic, paper-based device

## Abstract

In this article, we compare linear sweep anodic stripping voltammetry (LASV) and square-wave anodic stripping voltammetry (SWASV) for detection of a nano metalloimmunoassay. Two separate immunoassays were examined: a model assay, based on interactions between antibodies, and a sandwich assay for the heart failure marker NT-proBNP. In both cases, one antibody is linked to a magnetic microbead, and one is linked to a spherical Ag nanoparticle label. Electrochemical detection is carried out on a paper device. The three analytical figures of merit studied were the precision of the measurements, the calibration sensitivity, and the limit of detection (LOD). For the NT-proBNP assay, the results show that after optimization of the pulse amplitude and frequency of the potential input for SWASV, the detection efficiency is substantially higher compared to LASV. Specifically, the calibration sensitivity increased by up to ~40 fold, the average coefficient of variation decreased by ~40%, and the (LOD) decreased to 300.0 pM. Finally, for a model immunoassay, a ~10-fold decrease in the LOD was observed for SWASV compared to LASV.

## 1. Introduction

In response to stretching caused by increased blood volume, cardiomyocytes in the heart ventricles release prohormone brain natriuretic peptide (proBNP), which is subsequently enzymatically cleaved into brain natriuretic peptide (BNP) and N-terminal prohormone brain natriuretic peptide (NT-proBNP) before being released into the bloodstream [1]. Consequently, the concentration of NT-proBNP in the blood is used for diagnosing and evaluating the severity of heart failure, and for evaluating the efficacy of treatment regimens [2]. However, the concentration of natriuretic peptides can vary depending on comorbidities, age, and the race of the patient [3,4,5,6,7]. Accordingly, there is a demonstrated need for an inexpensive, quantitative sensor for monitoring NT-proBNP levels at home [8].

**Scheme 1:**
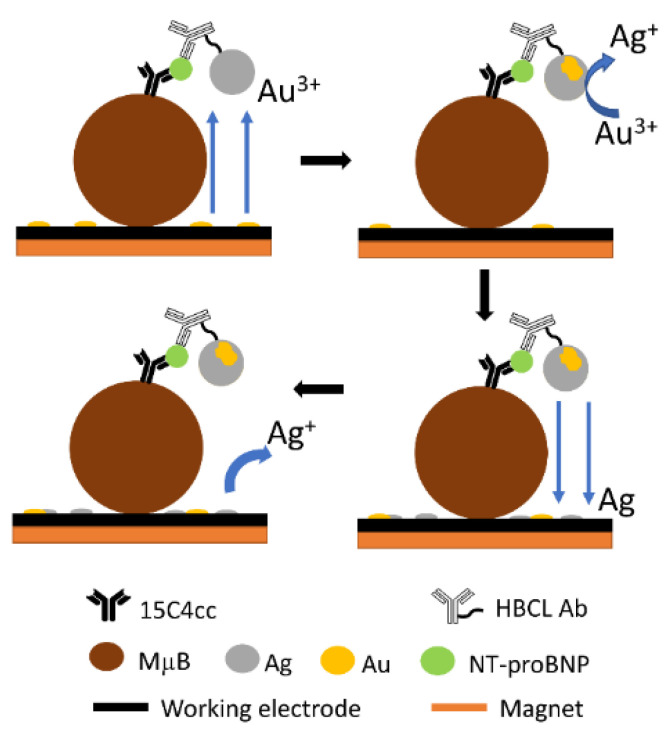
Schematic representation of the electrochemical detection process for the NT-proBNP metalloimmunoassay.

To address this need, we have been developing a low-cost diagnostic tool for the point-of-care quantification of NT-proBNP. The method is based on a metalloimmunoassay consisting of capture antibodies (Abs) conjugated to magnetic microbeads (MµB) and silver nanoparticle (AgNP)-labeled detection Abs. The device operates as shown in Scheme 1 [9,10]. First, after the NT-proBNP immunoassay is formed, it is preconcentrated onto a screen-printed electrode fabricated on a paper-based device and positioned over a magnet. Second, Au, pre-deposited on the working electrode (WE), is oxidized to yield Au^3+^. Third, Au^3+^ diffuses to AgNP and a process known as galvanic exchange (GE) takes place between Au^3+^ and the AgNP [10,11]. The GE process takes advantage of the difference in redox potential between two metals such that the less noble Ag (*E*^0^ = 0.79 V) spontaneously exchanges with more noble Au^3+^ (*E*^0^ = 1.52 V) to yield Au and Ag^+^_._ Fourth, the Ag^+^ resulting from the GE is electrodeposited onto the WE and, finally, electrochemical anodic stripping voltammetry (ASV) is used to quantify the amount of Ag deposited on the WE. The amount of charge collected during ASV is correlated to the concentration of NT-proBNP in the sample. 

The performance of a diagnostic device or analytical method is directly dependent on the goodness of its calibration curve [12]. Some of the most important characteristics of a calibration curve are its calibration sensitivity, limit of detection (LOD), and precision. Calibration sensitivity is defined as the change in signal magnitude as a function of the change in concentration of the target [13]. Here we have defined LOD as the smallest amount of substance in a sample that can be distinguished from the blank. Finally, precision is related to the random analytical error and is quantified by the coefficient of variation (COV): the lower the COV, the higher the precision [14]. Accordingly, improving these parameters results in a more robust assay. 

Square-wave voltammetry (SWV) is frequently used for electroanalytical methods requiring lower detection limits than are achievable by the more common methods of amperometry or cyclic voltammetry [15,16]. In SWV, the potential is modulated using a square-shaped potential pulse superimposed on a linear potential ramp [15,17], and the current is sampled at the end of each potential pulse to minimize the charging current. The applied potential in SWV depends on three characteristics of the square wave: frequency (*f*), amplitude (*E*_sw_), and potential increment (Δ*E*). This high degree of tunability makes it possible to select parameters that can be optimized for a particular analysis [18]. Thus, SWV has been used for studying and detecting many target analytes such as proteins [19], biomolecules [20,21], and pharmaceutical drugs [22,23].

Square-wave anodic stripping voltammetry (SWASV) has been used extensively to detect metal ions for environmental analyses. For example, Henry and co-workers used it to determine Pb^2+^ in contaminated soils [24]. Koley and coworkers used SWASV to detect Cu^2+^ in water samples [25]. As a final example, Banks and co-workers used SWASV to determine Pb^2+^ [26]. 

In this study, we have taken advantage of the flexibility of SWASV previously demonstrated for environmental samples to significantly improve the analytical metrics of our NT-proBNP bioassay compared to previous results that were based on linear anodic stripping voltammetry (LASV) [27,28]. Specifically, we have shown that the calibration sensitivity can be improved up to ~92-fold for a model composite (MC) and up to ~40-fold for the NT-proBNP assay itself compared to identical assays carried out by LASV. Additionally, the average COVs using SWASV are as low as 3.6% and 9.7% for the MC and NT-proBNP assays, respectively. These values can be compared to those obtained using LASV: 9.5% and 15.7% for MC and NT-proBNP assays, respectively. Finally, an LOD of 1.0 pM and 300.0 pM is obtained for MC and NT-proBNP assay, respectively, using SWASV compared to 10.0 pM and 550.0 pM obtained using LASV.

## 2. Experimental Section

### 2.1. Chemicals and Materials

All solutions were prepared using deionized (DI) water (>18.0 MΩ-cm, Milli-Q Gradient System, Millipore, Burlington, MA, USA). Phosphate buffered saline (PBS) pH 7.4 (P3813), superblock blocking buffer containing PBS (SBB) (cat. no. 37515), siliconized low-retention microcentrifuge tubes, HAuCl_4_, KNO_3_ and, Whatman grade 1 chromatography paper (180 μm thick, 20 cm × 20 cm sheets, linear flow rate of water = 0.43 cm/min) were purchased from Fisher Scientific (Pittsburgh, PA, USA). All PBS concentrations were 1X. The 5 kDa methyl polyethylene glycol-thiol (mPEG-SH) was obtained from Nanocs (New York, NY, USA). Sodium meta-periodate (NaIO_4_) was obtained from Thermo Scientific (Grand Island, NY, USA). Citrate-capped AgNPs (nominal diameter = 20 nm) were purchased from nanoComposix (San Diego, CA, USA). NT-proBNP, monoclonal immunoglobulin G anti-NT-proBNP 13G12cc detection Ab, and nonbiotinylated monoclonal immunoglobulin G anti-NT-proBNP 15C4cc capture Ab were obtained from HyTest (Turku, Finland).

Amicon Ultra 0.5 mL centrifugal filters (10 K) were purchased from Millipore-Sigma (Taunton, MA, USA). Conductive carbon paste (Cl-2042) was purchased from Engineered Conductive Materials (Delaware, OH, USA). Cylindrical neodymium magnets (1/16 in. × 1/2 in., N48) were purchased from Apex Magnets (Petersburg, WV, USA). All chemicals and reagents were used without further purification unless otherwise specified.

### 2.2. Instrumentation

A Sorvall Legend Micro 21R centrifuge from Thermo Scientific (Grand Island, NY, USA) was used for washing and separation steps during bioconjugation. A tube revolver (cat. no. 88881001), also from Thermo Scientific, was used or incubation steps during the conjugation of Abs to MμBs. A Mini Vortexer (945300) from VWR International (Radnor, PA, USA) was used to briefly mix solutions while a BioShake iQ from Quantifoil Instruments GmbH (Jena, Germany) was used for incubation steps during bioconjugation.

### 2.3. Electrochemistry

Electrochemical measurements were performed using a CH Instruments model 760B electrochemical workstation (Austin, TX, USA). The fabrication of paper electrodes and electrodeposition of Au onto the WE are provided in the Supporting Information and are based on methods described in a previous publication [10]. AgNP labels were detected using a previously published protocol [10,11,27,29]. Briefly, the potential of the WE was stepped from 0.0 to 0.8 V (all potentials are vs. a carbon quasi-reference electrode (CQRE)) for 12.0 s to oxidize pre-deposited Au^0^ to Au^3+^ to initiate the GE process. Ag^+^ formed during GE was then electrodeposited onto the WE by stepping down potential from 0 to −0.7 V for 50.0 s. These two steps were repeated one more time. Next, LASV was applied wherein the potential was swept from −0.7 to 0.2 V at a scan rate of 50 mV/s. Finally, an ASV (SWASV or LASV) was applied wherein the potential was again swept from −0.7 to 0.2 V to oxidize Ag from the WE. The charge collected for LASV, or the peak current for SWASV, was determined and correlated to the concentration of the target. 

### 2.4. Preparation of the AgNP-Ab Conjugates

A previously published protocol was used to conjugate 13G12cc Ab with AgNPs [30]. Briefly, the 13G12cc Ab was bioconjugated to a heterobifunctional cross-linker (HBCL) which was then added to 500 µL of AgNPs (4.9 × 10^11^ AgNPs/mL) and incubated for 1 h. Following incubation, the AgNP surface was back-filled with mPEG-SH for 20 min at 600 rpm in a bioshake at room temperature (RT, 22 ± 3 °C). The conjugate was then centrifuged for 30 min at 16,600× *g* at 4°C to remove any excess material. Finally, the remaining bioconjugate was resuspended in 300 µL of SBB. This conjugate will be referred to as the AgNP−Ab conjugate for this study.

### 2.5. Preparation of the MµB-Ab Conjugates

For the MC, the biotinylated SAb was conjugated to streptavidin-coated MµBs using the protocol provided by the manufacturer [31]. Specifically, 100 µL of MµBs (~7−10 × 10^9^ MµBs/mL) were aliquoted and washed using magnetic separation wherein the MµBs were collected on the wall of a microcentrifuge tube with a neodymium magnet, the supernatant was removed, and the conjugate was resuspended in PBS and washed again. This process was carried out three times. Next, 40.0 µL of 6.67 µM SAb were added to the tube and the resulting solution was incubated for 30 min at 30 rpm at RT using the tube revolver. Following conjugation, the MµBs were washed five times using magnetic separation with 100 µL of PBS and then resuspended in a final volume of 100 µL of 1% BSA *w*/*v* in PBS. The resulting conjugate will be referred to as MµB-SAb.

For the NT-proBNP assay, the 15C4cc capture Ab was biotinylated using a kit (ThermoFischer, Cat. No. 90407) and the protocol provided by the manufacturer [32]. Next, a similar procedure as described for the MµB-SAb was used to conjugate modified 15C4cc to the streptavidin-coated MµBs. Specifically, 20.0 µL of the 6.67 µM biotinylated 15C4cc capture Ab were incubated with 50 µL of the streptavidin-coated MµBs for 1 h at 30 rpm at RT on the tube revolver followed by washing using magnetic separation. The resulting product is referred to as the MµB-15C4cc conjugate.

### 2.6. Formation of Metalloimmunoassays

After preparing the MµB-SAb, MµB-15C4cc, and AgNP−Ab conjugates, two different metalloimmunoassay were prepared: the MC assay and the NT-proBNP assay. The MC assay was formed by conjugating MµB-SAb and AgNP-Ab (i.e., AgNP-13G12cc) via an interaction between the two Abs: 13G12cc and SAb. Specifically, 16.0 µL of the as-prepared MµB-SAb were added to 100 µL of the desired concentration of AgNP-Ab and then incubated for 30.0 min in the tube revolver at 30 rpm. The MC was then washed with 1% BSA *w*/*v* in PBS solution five times using magnetic separation and finally resuspended in 16.0 µL of PBS. 

A stepwise conjugation approach was used for the NT-proBNP assay. More specifically, this assay was formed in an SBB-blocked microcentrifuge tube as follows. First, 8.0 µL of the MµB-15C4cc conjugate was placed in the tube along with 50 µL of a desired concentration of NT-proBNP in SBB. These components were then incubated for 30 min at 30 rpm at RT. Next, the partially formed assay was washed three times using magnetic separation with SBB solution to remove unbound NT-proBNP. Finally, 50 µL of the AgNP-Ab was added. This mixture was again incubated for 30 min at 30 rpm in a tube revolver and was then washed using magnetic separation in SBB solution. The fully formed NT-proBNP assay was resuspended in a final volume of 8.0 µL of PBS. This conjugate will be referred to as MµB-NTproBNP-AgNP. 

For analysis, both the MC and the NT-proBNP assays (MµB-AgNP and MµB-NTproBNP-AgNP, respectively) were prepared similarly. First, 2.0 µL aliquots of the desired assay were combined with 48 µL of PBS in a tube to yield a final sample volume of 50.0 µL. This 50 µL sample was then transferred to the paper-based electrode, the fully formed assays were focused onto the WE (~30.0 s) by the magnet, and the remaining PBS was spread over all three electrodes to establish an electrical connection. Finally, the electrochemical procedure was performed as discussed earlier.

## 3. Results and Discussion

### 3.1. Electrochemical Analysis of the Current Signal as a Function of SWASV Parameters

**Scheme 2:**
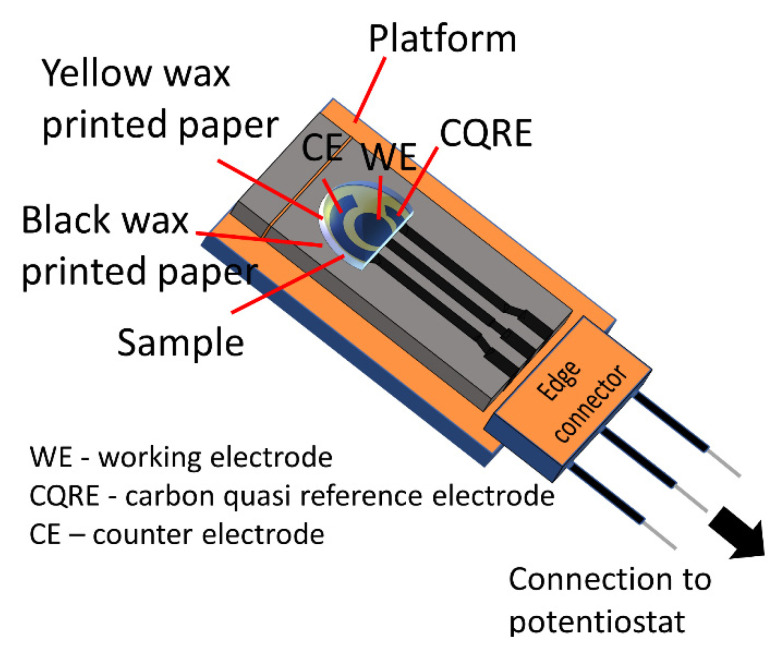
Schematic illustration of the wax-printed, paper-based device used for electrochemical detection.

The disposable paper-based devices used for electrochemical measurements were fabricated by stencil-printing carbon paste onto wax-patterned sheets of chromatography paper. Details are provided in the Supplementary Materials (SM) [10]. The device was placed over a platform with a magnet positioned underneath the WE (Scheme 2). 

To evaluate how SWASV parameters affect the current signal, we first studied their effect on the detection of the MC. As discussed in the Section 2, the MC was formed by conjugating MµB-SAb with AgNP-Ab. Subsequently, a 50 µL sample droplet (MC suspended in PBS) was placed onto the WE for ~30 s, and the remaining PBS was spread over all three electrodes to establish an electrical connection between the three electrodes. The AgNP concentration in these experiments was kept constant at 25.0 pM. 

Electrochemical detection of the assay proceeds through several steps, ultimately involving detection of the AgNP labels by SWASV. The resulting peak current is then used to determine the concentration of the MC (see Section 2 for details). From an electrochemical perspective, there are three SWASV parameters that can affect the peak current: *f*, *E*_sw_, and Δ*E*. It is known, however, that the impact of Δ*E* is small compared to *f* and *E*_sw_, and therefore we focused on the effect of the latter two parameters [18]. Accordingly, *f* was varied from 15 to 100 Hz with *E*_sw_ constant at 50 mV, and *E*_sw_ was varied from 15 to 100 mV with *f* constant at 50 Hz. The Δ*E* value was maintained at 4 mV in all experiments. 

**Figure 1:**
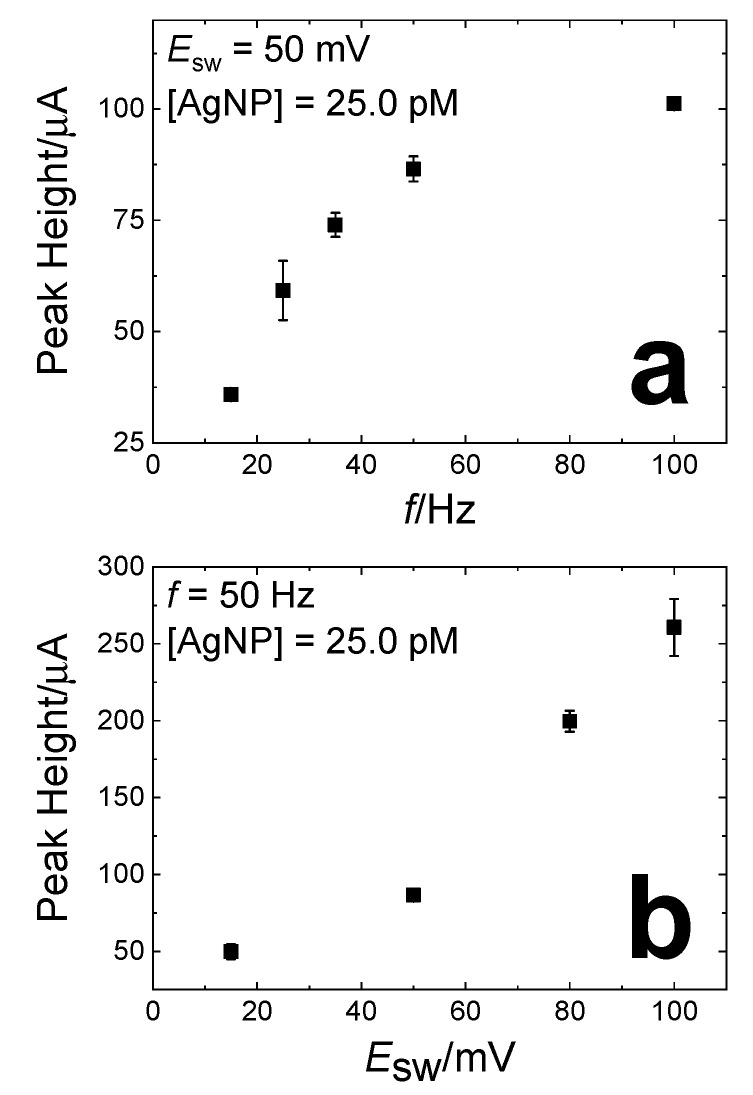
Variation of the SWASV peak height as a function of operational parameters. (**a**) The effect of increasing *f* at fixed *E*_sw_ (50 mV). (**b**) The effect of increasing *E*_sw_ at fixed *f* (50 Hz). Δ*E* was kept constant at 4.0 mV. Experiments were conducted on the MC using a fixed AgNP concentration of 25.0 pM. The error bars represent the standard deviation from the mean for three measurements obtained using independently fabricated paper electrodes.

Representative voltammograms for the MC ([AgNP] = 25.0 pM) obtained using different SWASV parameters are shown in Figure S1 in the ESI. The current peak heights of these voltammograms are plotted vs. *f* and *E*_sw_ in Figure 1. The results show that the current signal intensity increases upon increasing both *f* at fixed *E*_sw_ (Figure 1a) and *E*_sw_ at fixed *f* (Figure 1b). As has been found previously by others [18,33], we conclude that higher values of *f* and *E*_sw_ are desirable for maximizing peak current, and therefore these higher values were used for the analysis described in the next section.

### 3.2. Comparing Calibration Curves Obtained Using SWASV and LASV for the MC Assay

In this section, we examined the effect of SWASV on the calibration sensitivity and precision of the MC assay. Standard MC samples for producing calibration curves were prepared using AgNP concentrations ranging between 0 and 200 pM, and then electrochemical detection was carried out as discussed earlier. Three sets of experiments, each with a different final ASV step, were conducted to produce three calibration curves. The ASV steps used to generate these curves were as follows: (i) SWASV with *f* = 100 Hz and *E*_sw_ = 50 mV, denoted as SWASV50; (ii) SWASV with *f* = 100 Hz and *E*_sw_ = 100 mV, denoted as SWASV100; and (iii) LASV using a scan rate of 50 mV/s. Note that, in all cases, these final ASV scans followed a single LASV scan (see Section 2).

**Figure 2:**
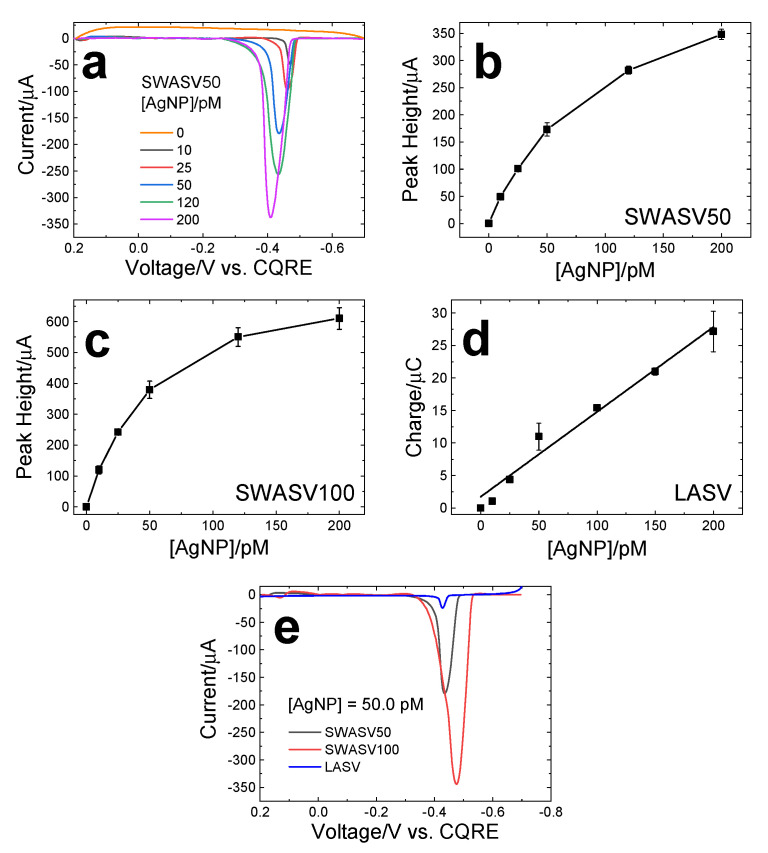
(**a**) Representative, baseline-corrected voltammograms for the SWASV50 set of experiments obtained using the MC and different concentrations of AgNP labels. Calibration curves for the (**b**) SWASV50, (**c**) SWASV100, and (**d**) LASV experiments. The error bars represent the standard deviation from the mean for 3–4 measurements obtained using independently fabricated paper electrodes. (**e**) Comparison of current peak height obtained using SWASV50, SWASV100, and LASV for a fixed AgNP concentration of 50.0 pM.

Representative voltammograms for the SWASV50 set of experiments are shown in Figure 2a. Qualitatively, these voltammograms indicate that the Ag oxidation current increases as a function of AgNP concentrations. The calibration curve in Figure 2b was generated by plotting the peak height of voltammograms like those in Figure 2a against the AgNP concentration. Similarly, two additional calibration curves were prepared for SWASV100 and LASV (Figure 2c,d, respectively; representative voltammograms for these plots are provided in Figure S2). The total charge for the LASV data (Figure 2d) was calculated by integrating the area under the peaks of voltammograms like those in Figure S2b. The most notable qualitative difference between the three calibration curves is that the curves for both SWASV50 and SWASV100 increase nonlinearly, while the plot for the LASV data is linear. 

To quantitatively compare the calibration curves in Figure 2, their calibration sensitivity and precision were determined. Because the LASV calibration curve is linear (Figure 2d), the calibration sensitivity is easily determined by measuring the slope of the curve. However, for the nonlinear SWASVs calibration curves (Figure 2b,c) the slope decreases with increasing analyte concentration. Therefore, we calculated a range of calibration sensitivities by measuring the highest and lowest slopes between adjacent data points. Additionally, for all three calibration curves, the COVs were calculated by averaging the COVs for the individual data points.

Table 1 shows the results of the foregoing measurements. Compared to LASV, the calibration sensitivity is improved by up to ~92-fold for SWASV100. Likewise, the COV for LASV is about 2.5 times as high as that of SWASV50. These results demonstrate the superior calibration sensitivity and precision of the SWASV technique.

To provide a direct comparison of the signal intensity for the SWASV50, SWASV100, and LASV methods, we plotted voltammograms for each technique at a fixed concentration of the MC ([AgNP] = 50.0 pM, Figure 2e). The data indicate that the SWASV100 method yields a peak current that is ~22 times higher than the LASV method. The SWASV50 voltammogram has an intermediate peak height (173 ± 12 pM). These results demonstrate the superior signal magnitude for SWASV vs. LASV.

### 3.3. Using SWASV for the NT-proBNP Assay

The previous section showed that the SWASV technique can lead to a superior calibration sensitivity and better precision for the detection of the MC. Accordingly, we applied SWASV for the detection of NT-proBNP, which is the primary focus of our sensor-related studies. 

The NT-proBNP sandwich assays were prepared as shown in Scheme 1 and discussed in the Section 2. The conjugated sample was then transferred to the paper device where the same electrochemical process used to detect the MC (Figure 2) was carried out. As for the MC case, we prepared three calibration curves corresponding to SWASV50, SWASV100, and LASV for the NT-proBNP assay. In all cases, these final ASV scans followed a single LASV scan (as for the data in Figure 2).

**Figure 3:**
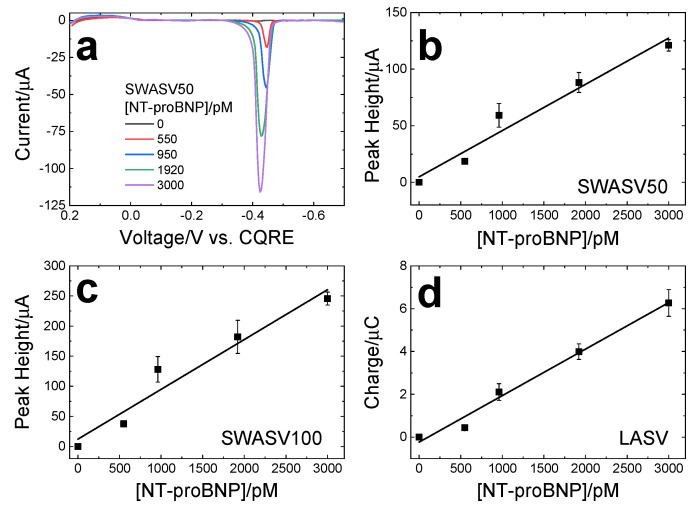
(**a**) Representative, baseline-corrected voltammograms for the SWASV50 set of experiments obtained for the NT-proBNP metalloimmunoassay and different concentrations of AgNP labels. Calibration curves for the (**b**) SWASV50, (**c**) SWASV100, and (**d**) LASV experiments. The error bars represent the standard deviation from the mean for 3–4 measurements obtained using independently fabricated paper electrodes.

Representative voltammograms for the SWASV50 set of experiments for standard samples of NT-proBNP conjugates are shown in Figure 3a. Qualitatively, these voltammograms indicate that the Ag oxidation current increases as a function of NT-proBNP concentration. The calibration curve in Figure 3b was generated by plotting the peak heights of voltammograms, like those shown in Figure 3a, against the NT-proBNP concentration. Following the same procedure as for the MC analysis, two additional calibration curves were prepared for SWASV100 and LASV (Figure 3c,d, respectively; the original data for these plots is provided in Figure S3). 

To quantitatively compare the data for the three calibration plots in Figure 3, we determined the calibration sensitivity and COV. Specifically, the calibration sensitivities were determined by measuring the slopes of the lines, and the COVs were calculated by averaging the COVs for the individual data points. 

Table 2 shows the results of these measurements. Two aspects of the data are noteworthy. First, the SWASV100 data reveal up to ~40 times improvement in calibration sensitivity compared to the results for LASV. Second, the COV data for all three ASV methods are roughly the same. Note, however, that samples having NT-proBNP concentrations lower than 550.0 pM could not be distinguished from the blank, even after using the SWASV method of detection. As we will show in a forthcoming manuscript, however, appropriately shaped AgNP labels can lower the LOD for the NT-proBNP assay into the risk stratification range for heart failure of 53 pM to 590 pM [29].

One final point, the maximum allowable COV of an assay for NT-proBNP detection is 10%. This can be calculated using the biological variation data provided by Westgard et al. [34]. As reported in Table 2, the LASV method produces an overall COV that is higher than this maximum allowable limit. For the SWASV50 method, however, COV is just below 10%. This is in addition to a significant enhancement in calibration sensitivity. Therefore, SWASV50 is the most appropriate parameter set for the measurement of NT-proBNP using the GE/ASV process. 

### 3.4. Improving the Limit of Detection

In this section, we report the LOD for MC and NT-proBNP assays carried out using SWASV and LASV. We define LOD as the lowest AgNP concentration that produces a current peak that can be differentiated from the blank. Because SWASV is considered to be a more sensitive method than LASV, and because we observe much higher currents for SWASV compared to LASV (for a fixed AgNP concentration, Figure 2e), we hypothesized that SWASV would yield a lower LOD than LASV. The results showed, however, that the LODs for the MC assay for both SWASV and LASV are ~10.0 pM (see Figure 2a for SWASV50, and Figure S2 for SWASV100 and LASV). Similarly, LOD for the NT-proBNP assay using both LASV and SWASV remained the same (~550.0 pM). We address this issue next. 

As discussed earlier and summarized by Scheme 1, Ag^+^ is produced during the GE process, and then it is electrodeposited onto the WE as zerovalent Ag. The amount of electrodeposited Ag is measured by applying two consecutive ASV scans (the abbreviation ASV refers to either SWASV or LASV). In all cases, the first of those two scans is always LASV, and the second scan is either LASV or SWASV depending on the objective of the experiment. The analytical signal is always determined from the voltammogram of the second ASV scan. This is because the voltammetric peaks arising from the first scans tend to be small and irreproducible compared to those of the second scans. We have previously speculated on possible explanations for this observation [11]. 

In the present case, however, we considered the possibility that for very low concentrations of AgNP labels, a more sensitive technique, such as SWASV, could lead to a lower LOD using just the first ASV scan. Accordingly, we used a single SWASV scan to gauge the effectiveness of this approach for lowering the LOD. 

The MC having a concentration of AgNPs equal to 1.0 pM was prepared as discussed earlier. To compare the two methods, the experiment was carried out using either a single LASV scan or a single SWASV scan following Ag electrodeposition. The ASV steps were as follows: (i) SWASV100 (*f* = 100 Hz and *E*_sw_ = 100 mV) or (ii) LASV using a scan rate of 50 mV/s. Similarly, we also conducted an identical single-scan electrochemical analysis on NT-proBNP immunoconjugate using 300.0 pM NT-proBNP to analyze the LOD of the NT-proBNP assay

**Figure 4:**
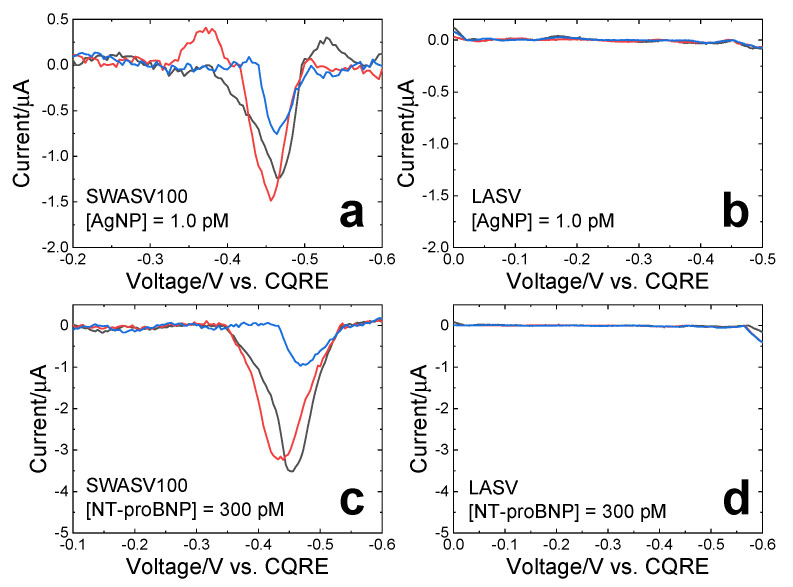
Baseline-corrected voltammograms for detection of the MC with 1.0 pM AgNP concentration using (**a**) SWASV100 or (**b**) LASV for the Ag stripping step. Baseline-corrected voltammograms for detection of the NT-proBNP immunoconjugate with 300.0 pM NT-proBNP concentration using (**c**) SWASV100 or (**d**) LASV for the Ag stripping step. The electrochemical detection sequence was identical to that of the experiments shown in Figure 2 up to and including the Ag electrodeposition step. Subsequently, only one final SWASV100 or LASV scan was applied. Three independently fabricated electrodes were used for each experiment.

The results for the foregoing experiments are shown in Figure 4. The voltammograms for three independently performed SWASV100 experiments for the MC assay are shown in Figure 4a. A peak height of 1.1 ± 0.2 µA is observed for a 1.0 pM concentration of AgNPs. When the same experiment is carried out using just one LASV scan, no detectable current peak is observed (Figure 4b). Likewise, a 300.0 pM NT-proBNP sample can be detected using a single SWASV100 scan (Figure 4c, average peak height = 2.5 ± 1.4 µA) whereas no peak was observed when a single LASV scan was applied (Figure 4d). Clearly, the LOD for the SWASV100 method is at least ten-fold and two-fold lower than for the LASV method for MC and NT-proBNP immunoconjugate, respectively, in this single-scan case.

## 4. Conclusions

The goal of this study was to improve the detection efficiency for the NT-proBNP metalloimmunoassay. In previous reports, we used only LASV for detection, but here we compared LASV and SWASV. The results indicate that SWASV can significantly enhance the calibration sensitivity, LOD, and assay precision for both the MC and NT-proBNP assays. The overall objective of our ongoing NT-proBNP sensor project has been to develop the electrochemical metalloimmunoassay method, implemented on a simple paper device, to a point where it overlaps the risk stratification range for heart failure of 53 pM to 590 pM of NT-proBNP concentration. This has involved tuning several aspects of the assay, including the shape of the AgNP labels [35], the electrochemical detection system [36], and, as shown here, the electrochemical methodology itself. All of this has brought us closer to our goal, and indeed we are now able to detect NT-proBNP in the middle of the risk stratification range for NT-proBNP. 

One final point merits mention. Although the focus of this study is NT-proBNP, the methodology is based on a simple sandwich immunoassay and an inexpensive paper-based device. In principle, therefore, this electrochemical detection approach can be applied to any target for which appropriate antibodies are available. Given the simplicity of the method and the picomolar detection limit, it seems likely that sensors for other targets could be developed.

## Figures and Tables

**Table 1:** Comparison of calibration sensitivity and COV for the MC assay carried out by SWASV and LASV.

Technique	Calibration Sensitivity	COV (%)
LASV	0.13 µC/pM	9.5
SWASV50	0.83–4.91 µA/pM	3.6
SWASV100	0.75–11.9 µA/pM	6.2

**Table 2:** Comparison of calibration sensitivity and COV for the NT-proBNP assay carried out by SWASV and LASV.

Technique	Calibration Sensitivity	COV (%)
LASV	0.0022 µC/pM	15.7
SWASV50	0.041 µA/pM	9.7
SWASV100	0.083 µA/pM	12.2

## Data Availability

The data is available upon reasonable request from the corresponding author.

## Supplementary Materials

The following supporting information can be downloaded at: https://www.mdpi.com/article/10.3390/bios12040203/s1, Fabrication of the paper-based device; Figure S1: ASV scans for different SWASV parameters for fixed AgNP concentration; Figure S2: Voltammograms for SWASV100 and LASV for MC detection; Figure S3: Voltammograms for SWASV100 and LASV for NT-proBNP detection.
