# Supplementary material for: Detection Efficiency of Ag Nanoparticle Labels for a Heart Failure Marker Using Linear and Square-Wave Anodic Stripping Voltammetry
Source: Biosensors (Basel). 2022 Mar 29;12(4):203. doi: 10.3390/bios12040203 (PMC9029172; doi:10.3390/bios12040203)
Supplement: Supplementary file 1 [file biosensors-12-00203-s001.zip › biosensors-1649299 supplementary.pdf]

biosensors

MDPI

Supplementary Information

Detection Efficiency of Ag Nanoparticle Labels for a Heart Failure Marker Using Linear and Square-Wave Anodic Stripping Voltammetry

Nikhil Raj and Richard M. Crooks *

Department of Chemistry, The University of Texas at Austin, 105 E. 24th Street, Stop A5300, Austin, TX 78712-1224, USA; nikhil.raj@austin.utexas.edu
* Correspondence: crooks@cm.utexas.edu

Fabrication of paper-based devices. A slight modification of a previously published procedure was used to fabricate the electrodes.[1] First, wax was printed on chromatography paper using a Xerox ColorQube 8570DN printer. Following printing, the wax was melted through the thickness of the paper by placing it in an oven at 120 °C for 30.0 s. Next, the back of the wax-printed chromatography paper was glued to photopaper to improve rigidity, and then it was cut into a rectangular shape (2.0 cm × 5.0 cm). A CAD drawing of the stencil for defining the 3.0 mm-diameter disk-shaped working electrode (WE), hook-shaped carbon quasi-reference electrode (CQRE), and counter electrode was created using CorelDRAW (Ottawa, ON). The stencil was cut into a thin plastic sheet of transparency film using an Epilog laser engraving system (Zing 16). Finally, the stencil was placed over the wax printed device and the electrodes were printed through the stencil using conductive carbon paste. The device was left to dry in the air for 14 h.

The WE was further modified with electrodeposited Au. This step was performed on-chip and was carried out by placing a 50.0 μL droplet of a solution consisting of 6.0 mM Au^{3+} and 0.10 M KNO_3 onto the electrode region of the sensor. Next, the potential of the working electrode was stepped from 0 to −0.60 V (unless otherwise stated, all potentials are vs. CQRE) for 2.0 s.[1] Finally, the electrode was rinsed twice with DI water and dried with a Kimwipe.

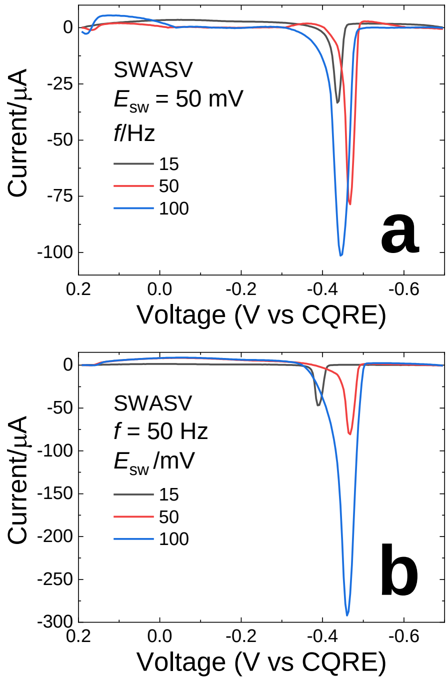

Figure S1. Representative voltammograms obtained for the MC showing (a) the effect of increasing f at fixed E_{sw} (50 mV) and (b) the effect of increasing E_{sw} at fixed f (50 Hz). ΔE was kept constant at 4.0 mV. In all cases, the concentration of AgNPs was 25.0 pM. The experimental conditions for these voltammograms were the same as for those described for Figure 1 in the main text. The position of the ASV peaks varies because the references electrode was a CQRE. The data were baseline corrected.

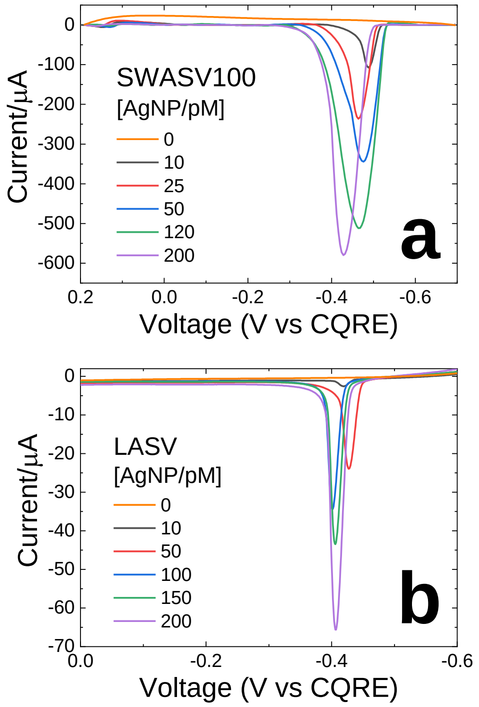

Figure S2. Representative voltammograms obtained for the MC using the (a) SWASV100 and (b) LASV detection methods. The concentration of AgNPs is provided in the legend. The experimental conditions and electrochemical seqeuence used for obtaining these voltammograms were the same as those discussed in the context of Figure 2 in the main text. The position of the ASV peaks varies because the references electrode was a CQRE. The baselines were corrected for the SWASV100 results.

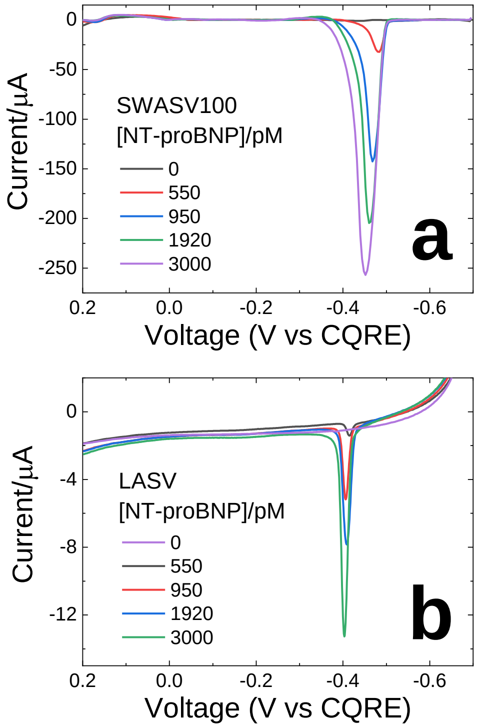

Figure S3. Representative voltammograms obtained using the (a) SWASV100 and (b) LASV methods for the indicated concentrations of NT-proBNP. The experimental conditions and electrochemical sequence used to obtain these voltammograms are provided in the main text in the context of Figure 4. The positions of the ASV peaks vary because the references electrode was a CQRE. The baseline was corrected for the SWASV100 results.

Reference

(1) Kogan, M. R.; Pollok, N. E.; Crooks, R. M. Detection of Silver Nanoparticles by Electrochemically Activated Galvanic Exchange. *Langmuir* **2018**, *34*, 15719–15726.
